# CO_2_ enrichment in greenhouse production: Towards a sustainable approach

**DOI:** 10.3389/fpls.2022.1029901

**Published:** 2022-10-21

**Authors:** Anran Wang, Jianrong Lv, Jiao Wang, Kai Shi

**Affiliations:** ^1^ Department of Horticulture, Zhejiang University, Hangzhou, China; ^2^ Yazhou Bay Science and Technology City, Hainan Institute, Zhejiang University, Sanya, China

**Keywords:** carbon dioxide, elevated CO_2_, controlled environment agriculture, horticulture, agro-industrial symbiosis system, carbon capture and utilization, CO_2_ assimilation

## Abstract

As the unique source of carbon in the atmosphere, carbon dioxide (CO_2_) exerts a strong impact on crop yield and quality. However, CO_2_ deficiency in greenhouses during the daytime often limits crop productivity. Crucially, climate warming, caused by increased atmospheric CO_2_, urges global efforts to implement carbon reduction and neutrality, which also bring challenges to current CO_2_ enrichment systems applied in greenhouses. Thus, there is a timely need to develop cost-effective and environmentally friendly CO_2_ enrichment technologies as a sustainable approach to promoting agricultural production and alleviating environmental burdens simultaneously. Here we review several common technologies of CO_2_ enrichment in greenhouse production, and their characteristics and limitations. Some control strategies of CO_2_ enrichment in distribution, period, and concentration are also discussed. We further introduce promising directions for future CO_2_ enrichment including 1) agro-industrial symbiosis system (AIS); 2) interdisciplinary application of carbon capture and utilization (CCU); and 3) optimization of CO_2_ assimilation in C_3_ crops *via* biotechnologies. This review aims to provide perspectives on efficient CO_2_ utilization in greenhouse production.

## Introduction

**Figure 1:**
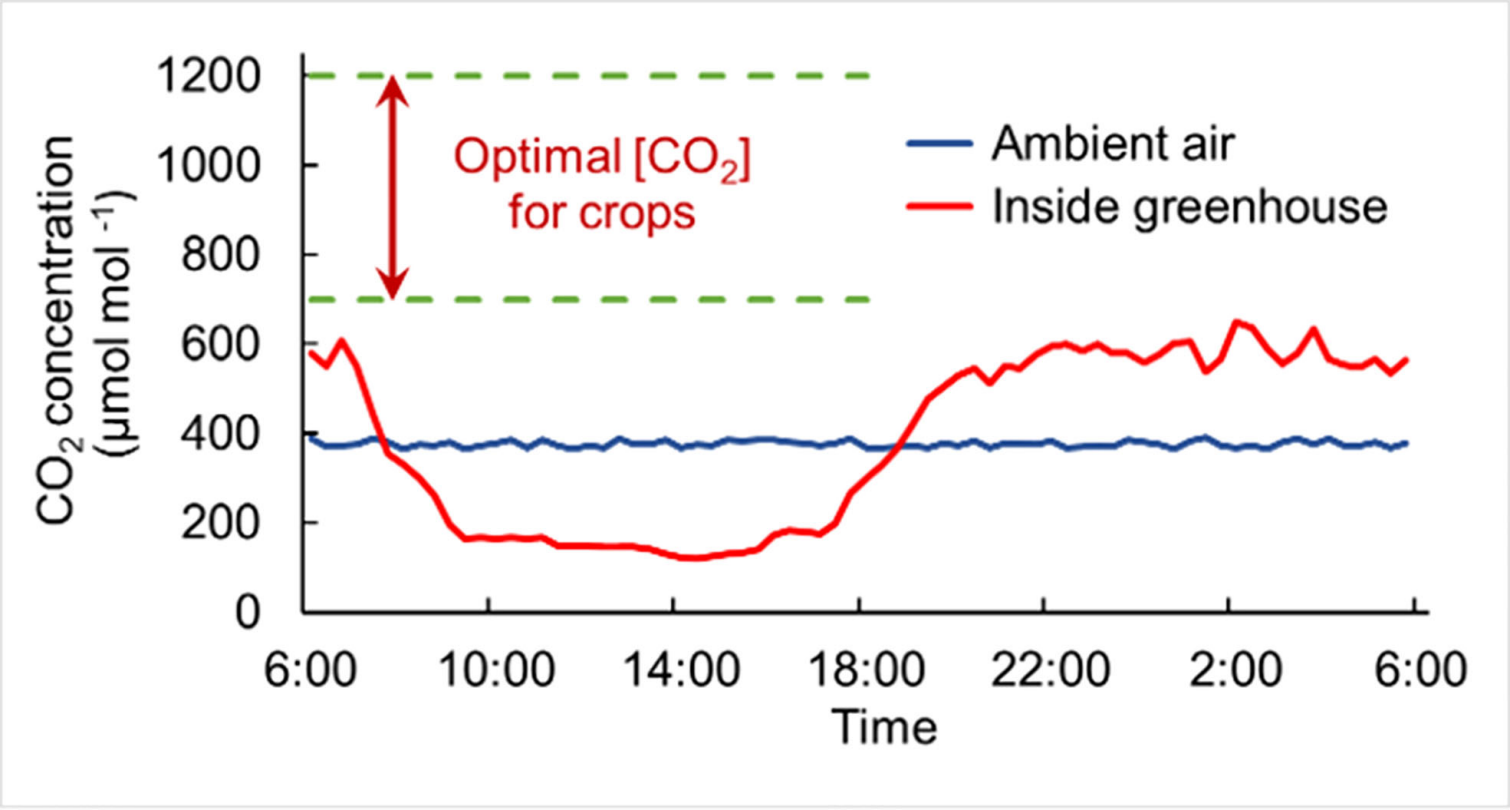
Schematic diagram of CO_2_ deficiency in greenhouse production. CO_2_ in the greenhouse (red curve) accumulates at night due to crop respiration, while the CO_2_ concentration decreases sharply after light exposure due to CO_2_ absorption by crops through photosynthesis, which is far below the crop demand (the range within green dotted lines), almost the whole daytime. The blue curve represents the CO_2_ level in the ambient air outside. The data for plotting the line graphs refer to the text in Zhang et al. (2014); Noctor and Mhamdi (2017); Dong et al. (2018); Li et al. (2018), and figures in Sánchez-Guerrero et al. (2005) and Kläring et al. (2007).

Food security requires greater and more consistent crop production against a backdrop of climate change and population growth (Bailey-Serres et al., 2019). Greenhouses offer solutions for protecting crops from extreme weather events and provide more suitable conditions for crop growth than open field cropping (Syed and Hachem, 2019). However, crops grown in greenhouses still suffer from multiple suboptimal conditions, one of which is frequent insufficient CO_2_ availability, limiting crop yield and quality (Poudel and Dunn, 2017). Due to a relatively airtight environment and crop uptake of CO_2_, the CO_2_ concentration in greenhouse drops to only 100~250 µmol mol^-1^ in the daytime, which is below the ambient CO_2_ level of 350~450 µmol mol^-1^ even with effective ventilation, and is far below the optimal concentration required for crop growth, 800~1000 µmol mol^-1^ (**Figure 1**; Pascale and Maggio, 2008; Zhang et al., 2014; Merrill et al., 2016).

Although various CO_2_ enrichment technologies have been developed for applications in protected cultivation for decades, CO_2_ concentration around the crop canopy is still a complex variate in modern agricultural environment control systems (**Table 1**; Linker et al., 1999; Kläring et al., 2007; Li et al., 2018). Unlike other environmental factors, CO_2_ needs to be controlled at a micro level (10^2^ ~10^3^ µmol mol^-1^), and is highly affected by ventilation, plant growth period, and weather (Wang et al., 2016; Li et al., 2018).

**Table 1:** Application examples of different CO_2_ enrichment technologies reported in scientific articles.

Enrichment technology	Principle	Crops	Treatment/control	Production effects of CO_2_ enrichment	References
**Compressed CO_2_** **injection**	Physical diffusion	Lettuce	700/400 ± 20 mol mol^-1^	Higher growth rates; Enhanced antioxidant capacity	Pérez-López et al., 2015
**Injection & Ventilation**	Physical diffusion	Cucumber	400-500/285-300 mol mol^-1^ average throughout the day	Increased fruit biomass; slightly effect on leaf area index	Sánchez-Guerrero et al., 2005
**Biogas burning**	Chemical reaction	Rose	800~2500 mol mol^-1^/ normal atmosphere	Enhanced fresh mass of cut flowers	Jaffrin et al., 2003
**Mixing baking soda** **with acid**	Chemical reaction	Mostly mentioned in reviews			Syed and Hachem, 2019
**Composting**	Biological activity	Tomato	800–900 mol mol^-1^/ not mentioned	Increased nutritional and sensory quality of fruits	Zhang et al., 2014

Currently, the ongoing global climate warming brings challenges to innovating and upgrading existing agricultural CO_2_ enrichment systems. Several key issues need to be addressed in terms of carbon reduction, such as direct CO_2_ emissions caused by an imbalance between CO_2_ supply, crop uptake, and ventilation operation (Vermeulen, 2014; Kozai et al., 2015), and resource consumption during the generation, transportation, storage of pure CO_2_ (Vermeulen, 2014). Moreover, the promotion of clean energy uses forces greenhouses that obtain CO_2_ from boiler heating systems to seek new enrichment solutions (Marttila et al., 2021).

Increasing endeavors are being devoted to improving CO_2_ enrichment in greenhouse production, while comprehensive articles on various techniques and solutions explored in production practices and scientific research are few. Here we review CO_2_ enrichment technologies and strategies applied in current greenhouse production or laboratory, focusing on their advantages and obstacles, and further summarize three promising directions for future agricultural CO_2_ enrichment, aiming to provide a sustainable approach to ensure food and climate security through agriculture.

## Effects of CO_2_ enrichment on greenhouse crops

**Figure 2:**
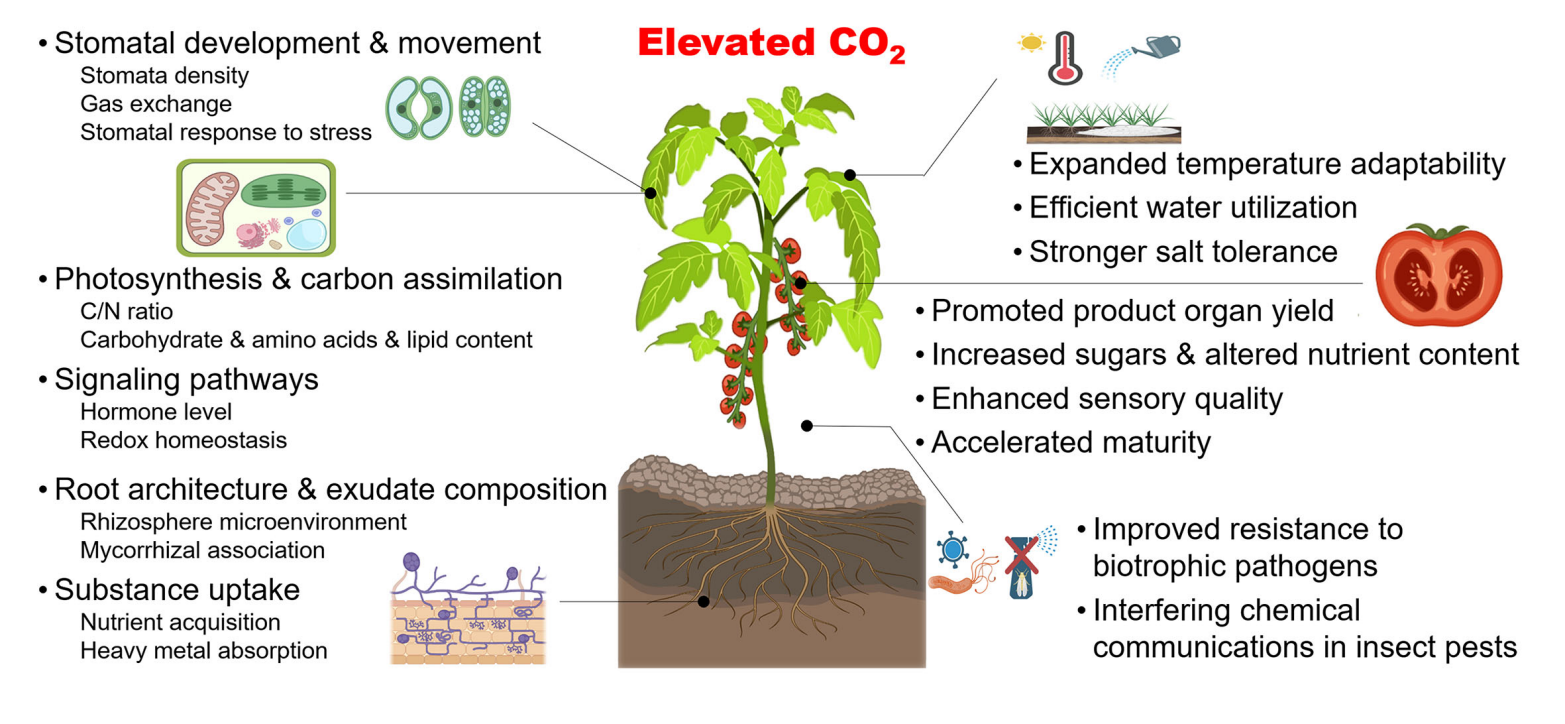
Schema illustrating effects of elevated CO_2_ on C_3_ crops. Elevated CO_2_ affects a series of plant biological processes (left) in C_3_ crops, including stomatal development and movement, photosynthesis, and carbon assimilation, signaling pathways, root development and exudate composition, and nutrient acquisition. Meanwhile, macro-production effects (right) can also contribute to promoted yield and quality, enhanced tolerance to abiotic stress and improved resistance to several biotic stresses, which invigorate efficient and safe agricultural production to a certain extent. Created with BioRender.com.

The crops grown in greenhouses are mainly C_3_ plants, such as tomatoes and cucumbers (Sage, 2017). Due to a lack of efficient mechanisms to cope with CO_2_ scarcity, C_3_ crops are more sensitive to changes in CO_2_ concentrations compared with C_4_ plants and CAM plants (Long et al., 2015). Importantly, C_3_ crops have a more positive response to increased CO_2_ concentrations (Ainsworth and Long, 2020). For instance, a moderate CO_2_ elevation of 550 ~ 650 µmol mol^-1^ improves the yield of various C_3_ crops by an average of 18% (Ainsworth and Long, 2020). Moreover, the CO_2_ concentration of around 1000 µmol mol^-1^ promotes the contents of soluble sugar and some nutrients of leafy, fruit and root vegetables by around 10% ~ 60% (Dong et al., 2018). As summarized in **Figure 2**, elevated CO_2_ is involved in a multitude of physiological activities in C_3_ crops including photosynthesis, signaling pathway, organ development, as well as the resistance to biotic and abiotic stresses, and CO_2_ enrichment further improves the yield and quality, and enhances the utilization efficiency of light and water (Zhang et al., 2015; Hu et al., 2021; Ahammed and Li, 2022). More detailed descriptions can be found in reviews by Xu et al. (2015); Dong et al. (2018); Kazan (2018); Ahammed et al. (2021); Poorter et al. (2021); Roy and Mathur (2021), and Chaudhry and Sidhu (2022).

## Current CO_2_ enrichment technologies

### Atmosphere ventilation

Ventilation allows exchanges of heat and CO_2_ inside and outside the greenhouse by means of natural ventilation with roof/side windows or forced ventilation (Ishii et al., 2014; Yasutake et al., 2017). Although ventilation can supply CO_2_ into greenhouses from atmosphere continuously, it is typically to regulate temperature preferentially, and an extra supply of CO_2_ is necessary for geographically cold regions with restricted ventilation (Stanghellini et al., 2008). Moreover, ventilation alone is not enough to maintain CO_2_ concentration around crops at an ambient air level (Pascale and Maggio, 2008), and crop yield is more heavily dependent on CO_2_ at a lower concentration (below 450 µmol mol^-1^) than a higher concentration (Vermeulen, 2014).

### Compressed CO_2_

The direct supply of compressed CO_2_ ensures a stable and clean airflow. However, due to the high market price and transportation cost, it is more commonly used as a complement to other techniques or in scientific research such as Free-Air CO_2_ Enrichment (FACE) (Sánchez-Guerrero et al., 2005; Allen et al., 2020). In addition, compressed CO_2_ needs to be equipped with devices for gas storage and pressure control that most often occupy some space in greenhouses (Kuroyanagi et al., 2014; Poudel and Dunn, 2017; Li et al., 2018).

### Carbonaceous fuel burning

When heating the greenhouse by combustion of natural gas, coal, biomass, and other carbonaceous fuels, CO_2_ generated during the processes can be delivered to crops or collected and stored for further use (Vermeulen, 2014). As a relatively effective approach to the reduction of carbon emissions and production costs, this technique is adopted widely in current greenhouse production (Dion et al., 2011; Marchi et al., 2018). Moreover, ventilation is often closed during heating, which ensures a better effect of CO_2_ enrichment (Kläring et al., 2007). A major limitation, however, is that for areas or seasons that do not require heating, burning fuel for CO_2_ is undesirable.

Given that the gas obtained from the combustion boiler carries too much heat and harmful gases, such as NO_x_, SO_2_ and CO, efficient procedures of cooling and purification are essentially required (Roy et al., 2014; Li et al., 2018). In addition, the time and dosage requirements often mismatch between CO_2_ and heat, resulting in a need for collection and storage devices and flow controllers of CO_2_ (Dion et al., 2011). Takeya et al. (2017) proposed a system to collect an appropriate amount of CO_2_ at night when the heating system is turned on and the gas can be released in the daytime when crops have a strong demand for CO_2_.

Notably, it is an increasingly urgent issue to replace carbonaceous fuels with clean energy to reduce carbon emissions, such as solar energy, hydrogen energy, geothermal energy, and even industrial waste heat (Vermeulen, 2014; Marttila et al., 2021). Meanwhile, the cost of production activities generating carbon emissions has increased drastically. Therefore, greenhouses obtaining CO_2_ from heating systems are facing a challenge to find alternative CO_2_ enrichment techniques (Vermeulen, 2014).

### Chemical reaction

The chemical reactions of bicarbonate (such as baking soda) with acid and the decomposition by direct heating are relatively cheap and fast to obtain pure CO_2_ quantitatively (Syed and Hachem, 2019). The CO_2_ production rate can be controlled theoretically while the operation in practice is complicated, and a large amount of CO_2_ generated out of control is wasted and can damage plants (Poudel and Dunn, 2017). Besides, ammonia bicarbonate is sometimes used as a raw material, which can produce by-products used as fertilizers. However, there is a threat of ammonia gas poisoning, so NH_3_ filtration is mandatory in such cases (Sun et al., 2016).

### Compost fermentation

Decomposition of carbon-rich agricultural wastes by microbial fermentation to release CO_2_ for crop production is considered a beneficial technology to increase production, reduce agricultural carbon emissions, and lower environmental pollution at the same time (Karim et al., 2020). But there are strict restrictions on C/N ratio, pH, temperature, materials, and other conditions (Jin et al., 2009; Karim et al., 2020). Technologies that use crop-residues and animal-manure composting (CRAM) to increase CO_2_ were developed to improve vegetable yield and quality (Jin et al., 2009; Karim et al., 2020). Secondary fermentation products could also be reused as a source of CO_2_ (Liu et al., 2021). Necessary measures should be taken to deal with several weaknesses in compost fermentation, such as 1) associated unpleasant odors; 2) threat of ammonia poisoning (Li et al., 2018); 3) unstable rate of generated CO_2_ (Karim et al., 2020); and 4) a larger space and more labor input requirements compared with other enrichment techniques (Tang et al., 2022).

## Control strategies of CO_2_ enrichment

The CO_2_-use efficiency (CUE), defined as the ratio of net photosynthetic rate to CO_2_ supply rate, suffers from various factors, such as excess supply, natural leakage, sensitive growth state of plants, and other environmental and biological components (Sánchez-Guerrero et al., 2005; Kuroyanagi et al., 2014; Li et al., 2018). The values of CUE in greenhouses are generally lower than 60%, which means that a considerable amount of CO_2_ is released into the ambient atmosphere (Kozai, 2013; Kuroyanagi et al., 2014). Thereby, numerous attempts have been made on control strategies of CO_2_ enrichment from various aspects to improve the CUE in the greenhouse.

### Spatial distribution

The uniformity of environmental elements contributes to a unified and efficient management of greenhouse cultivation, while the spatially uneven distribution of CO_2_ is universal in almost all greenhouse cultivation (Li et al., 2018). Due to the lack of air circulation and the relatively slow diffusion, CO_2_ concentration is extremely low around the canopy with high leaf density where CO_2_ is in most demand (Hidaka et al., 2022). Enrichment systems, with single-point outlet, make CO_2_ more uneven in space, resulting in great waste and failure to meet the production demand (Zhang et al., 2020). Thus, some conveying pipes with holes around the leaves need to be assembled. Hidaka et al. (2022) applied pipe-delivered crop-local CO_2_ enrichment in strawberry cultivation and achieved increased yield with CO_2_ supply savings. Another option is by means of internal airflow stirring devices, which is also feasible (Boulard et al., 2017; Syed and Hachem, 2019).

### Period setting

There are various modes in the period setting of CO_2_ enrichment, such as throughout the day and night, during the daytime, and only in the morning or nighttime. Enrichment throughout the day and night or the whole daytime is generally adopted in controlled chambers for experimental purposes (e.g., Mamatha et al., 2014; Hu et al., 2021). Apparently, it is high energy-consuming and carbon-emitting to elevate CO_2_ all day in production, especially since the carbon assimilation is typically most intense in the morning of the whole day (Xu et al., 2014). More critically, photosynthetic acclimation can occur with crops over prolonged periods of exposure to elevated CO_2_ (Wang et al., 2013). Thus, strategies of enriching CO_2_ only in the morning rather than all daytime have been explored. Treatments of elevating CO_2_ only in the morning promoted biomass accumulation and flower/fruit quality with no difference from enriching throughout the daytime in some cases (Caliman et al., 2009; Xu et al., 2014). However, another similar strategy of CO_2_ enrichment with intermittence was found to suppress the promotion of photosynthesis and yield in cotton, wheat, chrysanthemums, soybeans, and tomatoes (Mortensen et al., 1987; Bunce, 2012; Allen et al., 2020). Besides, the effects of nighttime CO_2_ enrichment are still unclear, which may be species- or cultivar-dependent (Baker et al., 2022).

### Concentration control

The concentration gaps between inside and outside the greenhouse (C_in_-C_out_) and the air exchange rate (dominated by ventilation) are two key factors affecting CUE, besides the crop intrinsic photosynthetic capacity (Kozai et al., 2015; Yasutake et al., 2017). When the setting C_in_ is higher, e.g., 1000 µmol mol^-1^, the CUE is less than 50% even in an unventilated greenhouse due to a massive leakage of CO_2_ (Kuroyanagi et al., 2014). Moderate control systems of CO_2_ enrichment based on crop absorption rate or C_in_-C_out_, with a CUE close to 100%, have been reported to improve the yield of cucumbers and tomatoes (Kläring et al., 2007; Kozai et al., 2015). Thus, it is a feasible and sustainable strategy to keep a moderate CO_2_ concentration slightly higher than the ambient concentration in the cultivation environment, e.g., 550 ~ 650 µmol mol^-1^, considering economic cost and environmental protection (Vermeule, 2014; Kozai et al., 2015). The resulting gaps in yield and quality compared with crops cultivated in optimal CO_2_ concentration might be alleviated by controlling other environmental conditions and imposing moderate environmental stresses (Kozai et al., 2015; Dong et al., 2018).

Notably, unlike the consistent conclusion of an increase in yield, the effects of elevated CO_2_ on crop quality are diverse (Dong et al., 2018), suggesting that the optimal CO_2_ concentration should be determined by specific production requirements rather than a constant value. Compared with ambient CO_2_ and a lower CO_2_ elevation (550 µmol mol^-1^), the synthesis of glucose and fructose are promoted under higher CO_2_ concentration (700 - 1000 µmol mol^-1^), while some amino acids and minerals are deceased (Högy and Fangmeier, 2009; Dong et al., 2018). The changes in health-promoting compounds and flavor substances under elevated CO_2_, such as flavonoids, lycopene, and ascorbic acid, carotene, are controversial in different vegetable crops, perhaps due to characteristics of different product organs and disturbance of synthesis processes by other environmental conditions (Mamatha et al., 2014; Dong et al., 2018; Hao et al., 2020).

## Directions for future CO_2_ enrichment

**Figure 3:**
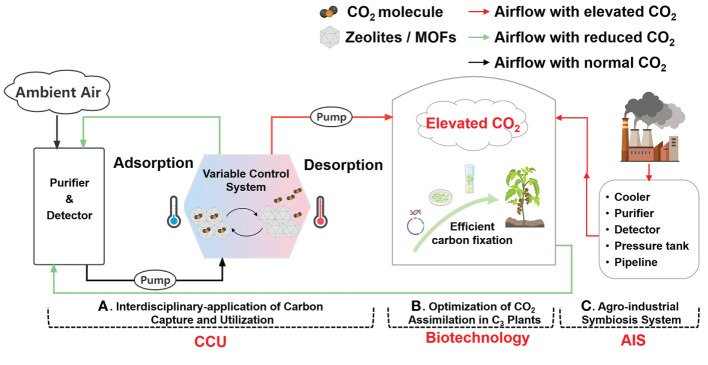
Overview of directions for future CO_2_ enrichment in greenhouse production. **(A)** Interdisciplinary application of carbon capture and utilization. Zeolites/MOFs as carbon adsorption materials can adsorb CO_2_ molecules from ambient air at low temperatures and desorb them at high temperatures to enrich CO_2_ into greenhouses. **(B)** Optimization of CO_2_ assimilation in C_3_ crops. Limited CO_2_ assimilation ability in C_3_ crops can be improved *via* biotechnological approaches including gene manipulation and genetic transformation. **(C)** Agro-Industrial Symbiosis system. A direct connection between the energy industry and greenhouses can be built through a pipeline network to convey CO_2_ and heat. Created with BioRender.com.

In addition to the challenge of increasing yields and improving quality, the global agricultural production system also faces tremendous pressure to reduce its carbon footprint to mitigate climate change. Even though photosynthesis of crops largely consumes CO_2_ as the endogenous driving force of agriculture, protected agriculture in various countries and regions is still a carbon emission-intensive process (Marttila et al., 2021; Northrup et al., 2021). Thus, taking full advantage of the crop ability of carbon fixation and combining the advantages of various disciplines should be a sustainable strategy to meet challenges in global food production and climate change simultaneously. In this regard, three novel and potentially feasible directions for future CO_2_ enrichment (**Figure 3**) are summarized and discussed as follows.

### Agro-industrial symbiosis system (AIS)

Burning fossil fuels and the operation of non-renewable energy-based industries are being restricted gradually due to their intensive contribution to the majority of global carbon emissions. Strategic management of the agricultural production system has the potential to provide beneficial contributions to the global carbon budget (Marchi et al., 2018; Northrup et al., 2021; Friedlingstein et al., 2022). Thus, a novel agro-industrial symbiosis system (AIS) of channeling industrial waste heat and CO_2_ to greenhouse productions through pipeline networks is proposed as a viable solution (Marttila et al., 2021). Compared with traditional AIS systems which only transfer heat, this system reduces carbon taxes related to CO_2_ emissions in industrial processes while increasing revenues of agricultural production (Marchi et al., 2018). Bottlenecks are the initial construction cost and design. The greenhouse needs to be within a limited distance (e.g., 10 km) of the factory, with a matching demand dosage of CO_2_; and the change of CO_2_ concentration during the delivery and purification of source gas needs to be considered (Vermeulen, 2014; Marchi et al., 2018).

### Interdisciplinary-application of carbon capture and utilization (CCU)

Carbon dioxide capture, utilization, and storage technologies (CCUS) are being vigorously researched. Compared with the huge cost and risk of leakage of carbon storage, converting CO_2_ into substances that people need, that is, carbon capture and utilization (CCU), is more attractive (Hepburn et al., 2019). Agriculture has an inherent advantage in this regard owing to the original demand for CO_2_. But there is a long way to go from now to real applications in agricultural production.

Physical adsorption, with lower energy consumption and milder reaction conditions, may be the most suitable for agricultural production among various methods of carbon capture including absorption solution, calcium looping, membrane technology and microalgal bio-fixation (Ben-Mansour et al., 2016). Target fluid molecules like CO_2_, can be selectively adsorbed through the huge surface area, specific pore structures, and ions inside the adsorbents (Zhou et al., 2021). Processes of reversible adsorption and desorption are controlled by changing conditions such as temperature and pressure (Ben-Mansour et al., 2016; Zhou et al., 2021).

There are two sources of CO_2_ capture: 1) industrial exhaust, which is confined and high in concentration; and 2) natural atmosphere, which is widespread and low in concentration. The latter, which is called direct air capture (DAC), is more challenging but also more practically meaningful (Maina et al., 2021). However, the desorption capacity especially required in agricultural production is often overlooked in studies on DAC (Bao et al., 2018). And though there are kinds of adsorbents with various properties for options, the adsorption and desorption capacities are often antagonistic (Zhou et al., 2021). Therefore, the suitable CCU material for agricultural production remains to be explored or transformed.

Most of the control methods of utilizing CCU materials for CO_2_ enrichment practices in agriculture production are based on variable temperature, as shown in **Figure 2**. Bao et al. (2018) used a water bath to control the temperature, and calculated that the cost of using 13X zeolite was close to that of the cheapest way of burning natural gas (halved because of the supply of heat), and can be lower considering the carbon tax. Araoz et al. (2021) developed conductive carbon tubes which could realize rapid temperature control of zeolite or metal-organic frameworks (MOFs) filled therein with voltage, providing an application model for greenhouse CO_2_ enrichment. Tang et al. (2022) reversed the temperature *via* a rotary regenerative wheel (RAW) loaded with carbon adsorbents, and analyzed the influence of gas flow, rotational speed, and other parameters on its CO_2_ enrichment performance.

Although related studies are only theoretically feasible with parts of the devices or designs, this direction deserves great attention owing to the carbon neutrality as no CO_2_ is freshly generated in the whole process. Besides, technological exploration of DAC is receiving increasing interest, and promisingly to play a breakthrough role in agriculture CO_2_ enrichment technology in the future (Bao et al., 2018).

The requirements of CCU in agriculture systems in future applications can be summarized as follows: 1) strong adsorption in ambient CO_2_ to provide sufficient pure CO_2_; 2) sustained desorption to generate a controllable flow of CO_2_; 3) low energy consumption in desorption or regeneration, such as lower temperature; 4) high adaptability to the agricultural environment with much water vapor and dust to ensure a stable effect in reusing (Wang et al., 2014; Bao et al., 2018).

### Optimization of CO_2_ assimilation in C_3_ crops *via* biotechnologies

Except for controlling the environment, modifying plant intrinsic carbon utilization efficiency by altering hereditary substances is a more efficient and revolutionary approach. The capability of CO_2_ fixation based on photosynthesis is limited especially in C_3_ plants (Yang et al., 2021). Great efforts have been put into the optimization of the photosynthesis system of C_3_ crops for decades (Raines, 2011). At present, the complicated mechanism of photosynthesis has been elucidated comprehensively and deeply (Long et al., 2015), preparing the foundation for improving plant carbon fixation *via* increasingly powerful biotechnologies.

As a crucial restriction enzyme of the C_3_ cycle, Rubisco catalyzes the binding of CO_2_ and its receptor ribulose 1,5-bisphosphate (RUBP), with a low activity and competitive dual functions of carboxylation and oxygenation (Long et al., 2015). The latter causes a loss of carbon and nitrogen fixation through photorespiration (Tcherkez, 2013). C_4_ plants have evolved a transcellular carbon concentration mechanism (CCM) that increases CO_2_ around rubisco, promoting photosynthetic carbon assimilation and reducing photorespiration significantly; and photosynthetic algae also have CCMs with different organelle structures (Zabaleta et al., 2012). Despite the tall order to introduce a whole CCM into C_3_ plants, both C_4_ plants and photosynthetic algae provide vital references and genetic materials for transforming the photosynthetic carbon fixation of C_3_ crops (Raines, 2011; Rae et al., 2017). For example, overexpression operations of phosphoenolpyruvate carboxylase (PEPC, an enzyme catalyzing the entry of bicarbonate into the C_4_ cycle), Sedoheptulose-1,7-bisphosphatase (SBPase, an enzyme involved in the regeneration of RUBP) and carbonic anhydrase (CA, an enzyme catalyzing conversion of intracellular CO_2_ into bicarbonate reversibly) from C_4_ plants or cyanobacteria, were all found effective to promote photosynthetic capacity in C_3_ crops (Köhler et al., 2017; Liu et al., 2021; Kandoi et al., 2022).

Naturally, what can be done to improve the CO_2_ utilization of C_3_ crops is far beyond modifying the C_3_ cycle, as listed below:

1) Searching for biological parts of metabolic processes from other organisms (Yang et al., 2021). For example, introducing photorespiratory bypasses from bacteria into rice increased photosynthesis by reducing energy losses in metabolism and releasing CO_2_ around Rubisco (Wang et al., 2020).

2) Improving the light utilization efficiency by expanding the absorption spectrum of light-harvesting pigments and the photosynthetic electron transport chain, which provide energy to the C_3_ cycle (Long et al., 2015).

3) Combining with computational modeling. Scientific prediction and analysis would accelerate the understanding and manipulation of complex life activities (Raines, 2011). e.g., Zhao et al. (2021) explained the underlying mechanism of mutual interference of enzymes in the C_3_ cycle by a dynamic systems model, and pointed out the requirement of balanced activities of enzymes to gain a greater photosynthetic efficiency, which would be further explored by an iterative design-built-test-learn approach (Patron, 2020).

## Outlook

Optimal CO_2_ concentration has great potential to further improve the yield and quality of agricultural products, especially nowadays when technologies of controlling temperature, light, water, and fertilizer are quite advanced and efficient. Meanwhile, with those intrinsic and emerging conundrums in current CO_2_ enrichment systems being overcome by multidisciplinary supports, efficient agricultural carbon utilization in greenhouse production would be a promising and sustainable advantageous solution to alleviating the pressure of food security and global warming.

In addition to those mentioned above, directions of improvement in the future agricultural CO_2_ enrichment can be expanded, such as exploring technologies suitable for open field production, and developing more sensitive sensors and more intelligent CO_2_ control models on period and concentration for greater CUE. Apart from photosynthesis, the important role of CO_2_ in plants also needs in-depth studies on mechanisms and improvements of photoadaptation and reduced nutrients requirement under elevated CO_2_.

## Author contributions

KS conceived the paper. AW wrote the paper. KS, JL, and JW revised the paper. All authors contributed to the article and approved the submitted version.

## Funding

This work was supported by the Natural Science Foundation of Zhejiang Province (LR19C150001), the National Natural Science Foundation of China (32172650), and the Starry Night Science Fund of Zhejiang University Shanghai Institute for Advanced Study (SN-ZJU-SIAS-0011).

## Conflict of interest

The authors declare that the research was conducted in the absence of any commercial or financial relationships that could be construed as a potential conflict of interest.

## Publisher’s note

All claims expressed in this article are solely those of the authors and do not necessarily represent those of their affiliated organizations, or those of the publisher, the editors and the reviewers. Any product that may be evaluated in this article, or claim that may be made by its manufacturer, is not guaranteed or endorsed by the publisher.
